# Supplementary material for: Restoring drifted electron microscope volumes using synaptic vesicles at sub-pixel accuracy
Source: Commun Biol. 2020 Feb 21;3:81. doi: 10.1038/s42003-020-0809-4 (PMC7035423; doi:10.1038/s42003-020-0809-4)
Supplement: Supplementary file 1 — Supplementary Information [file 42003_2020_809_MOESM1_ESM.pdf]

SUPPLEMENTARY INFORMATION

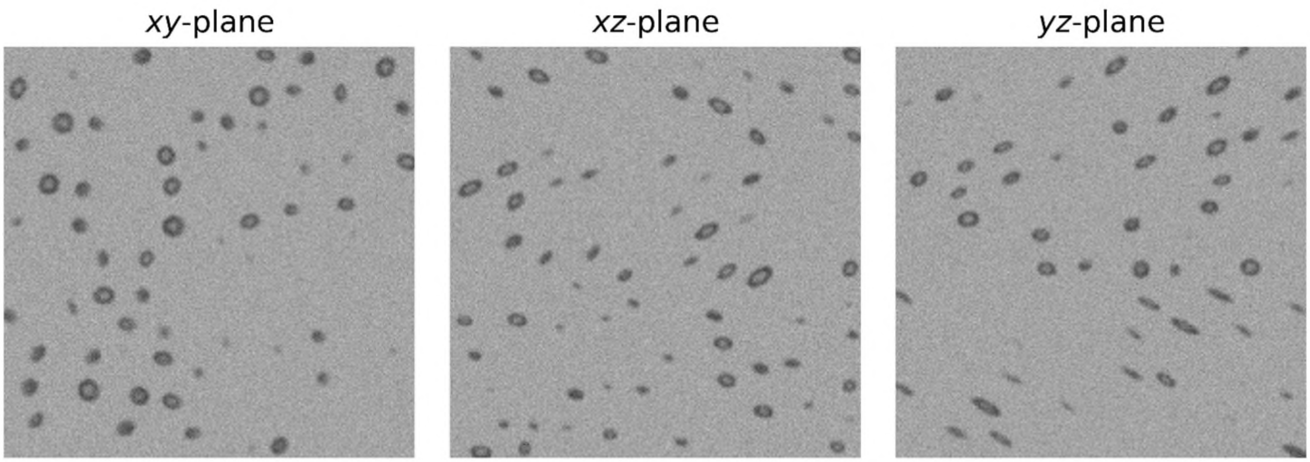

Supplementary Figure 1 | Example views of the synthetic dataset with varying drift. Synthetic vesicles can be seen drifted in different directions in the *xz* and *yz*-planes, an effect not noticeable in the classic *xy*-plane.

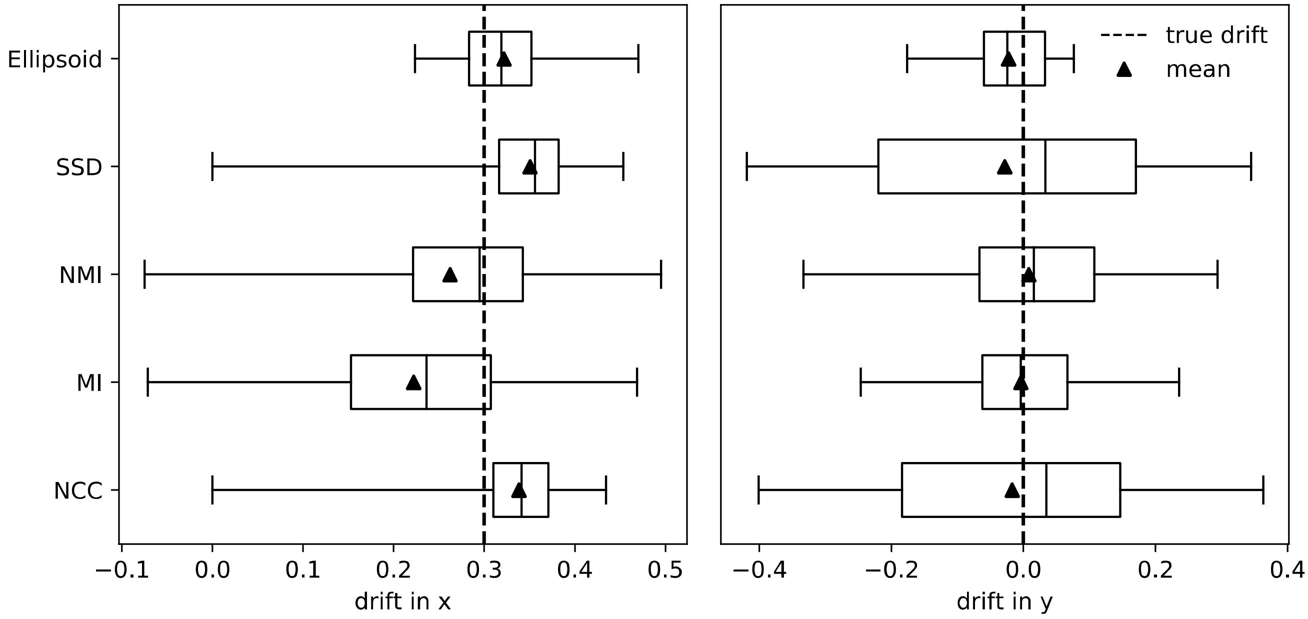

Supplementary Figure 2 | Comparison of the distribution of section drift estimates by the ellipsoid method and standard approaches on the synthetic dataset with a constant drift of (*x*, *y*)=(0.3, 0.0). Left: drift-estimate in the *x*-direction. The mean estimated drift for our ellipsoid method has both high accuracy and better precision than the comparison methods.

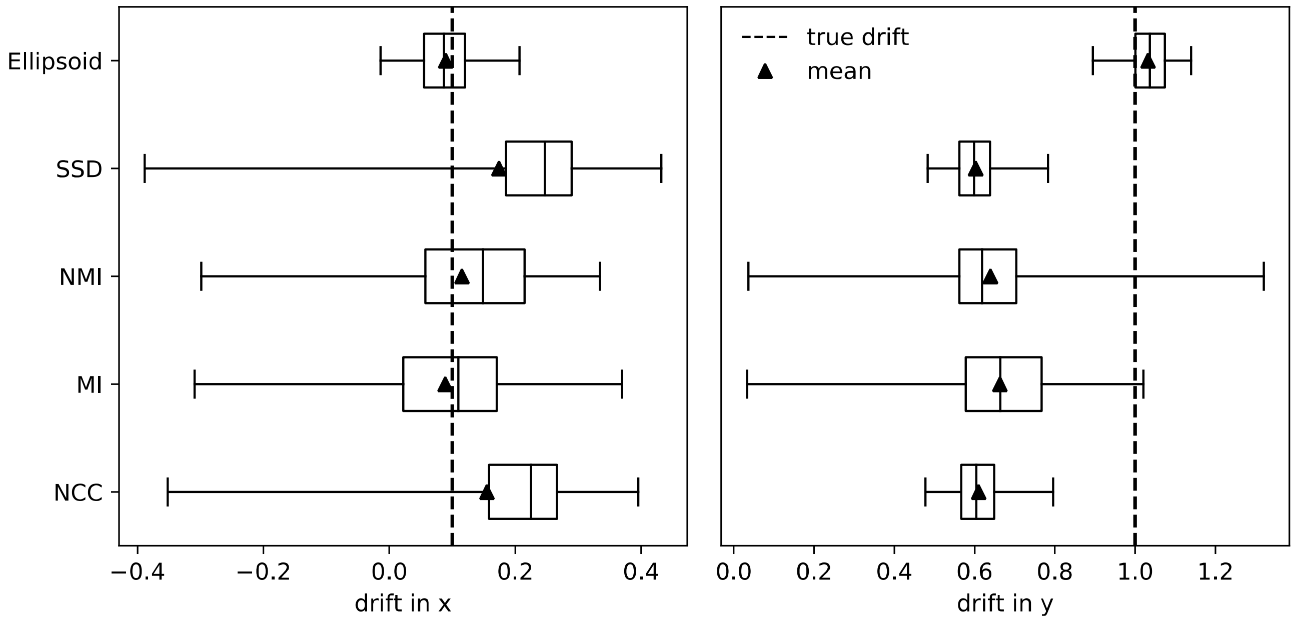

Supplementary Figure 3 | Comparison of the distribution of section drift estimates by the ellipsoid method and standard approaches on the synthetic dataset with a constant drift of (x, y) = (0.1, 1.0). Left: drift-estimate in the x-direction. For our method, the mean estimated drift is in close agreement with the true drift and display better precision than the comparison methods. The error of the standard, global methods appear to increase with increasing drift.

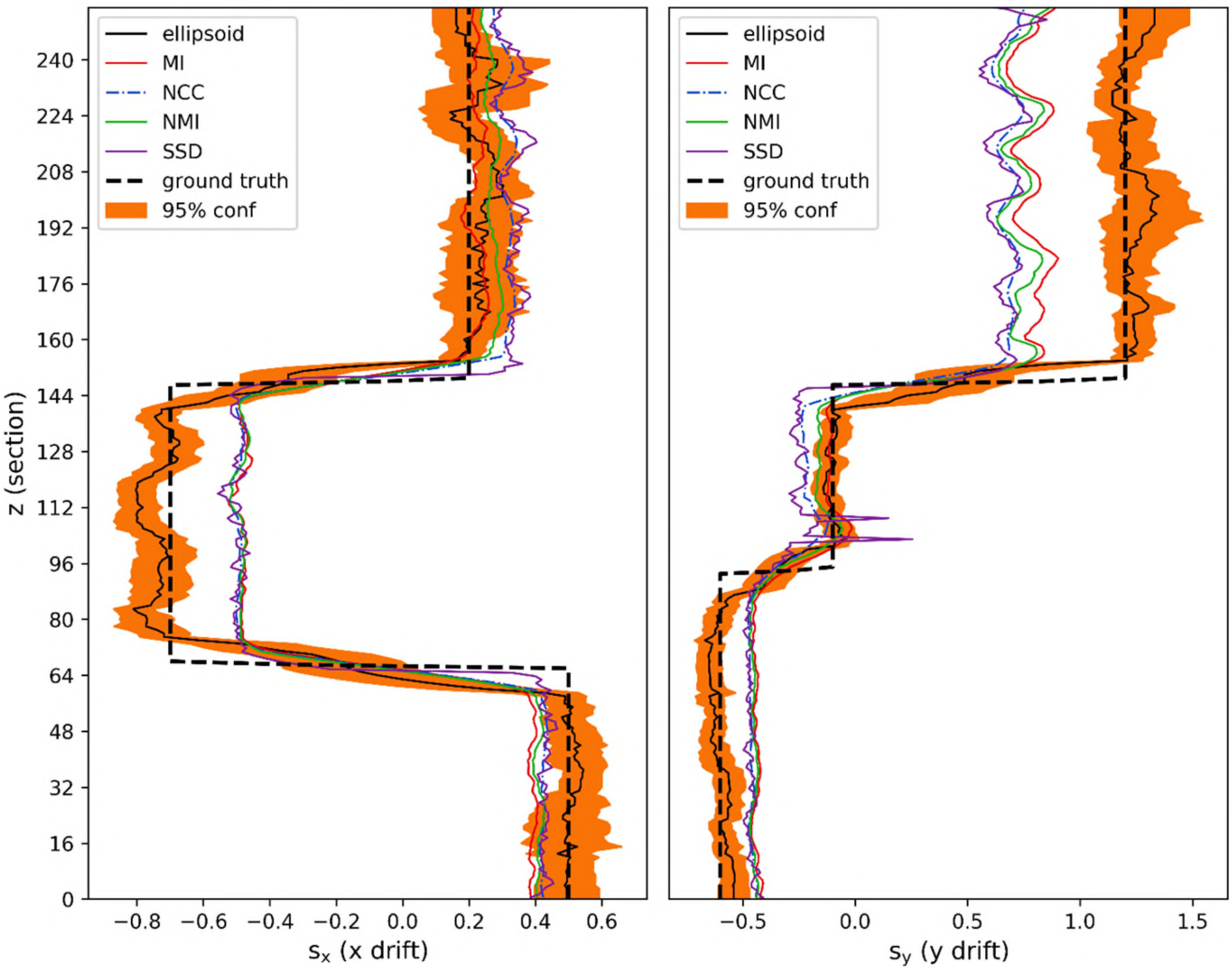

Supplementary Figure 4 | The complete drift estimate comparison in all 256 sections of the synthetic dataset with varying drift. All sequences except the one denoted *ellipsoid* were convolved with a box kernel of size 11 to counter the noise in those approaches to make visual inspection possible. The standard registration methods produce biased drift along both the *x* and *y*-axis.

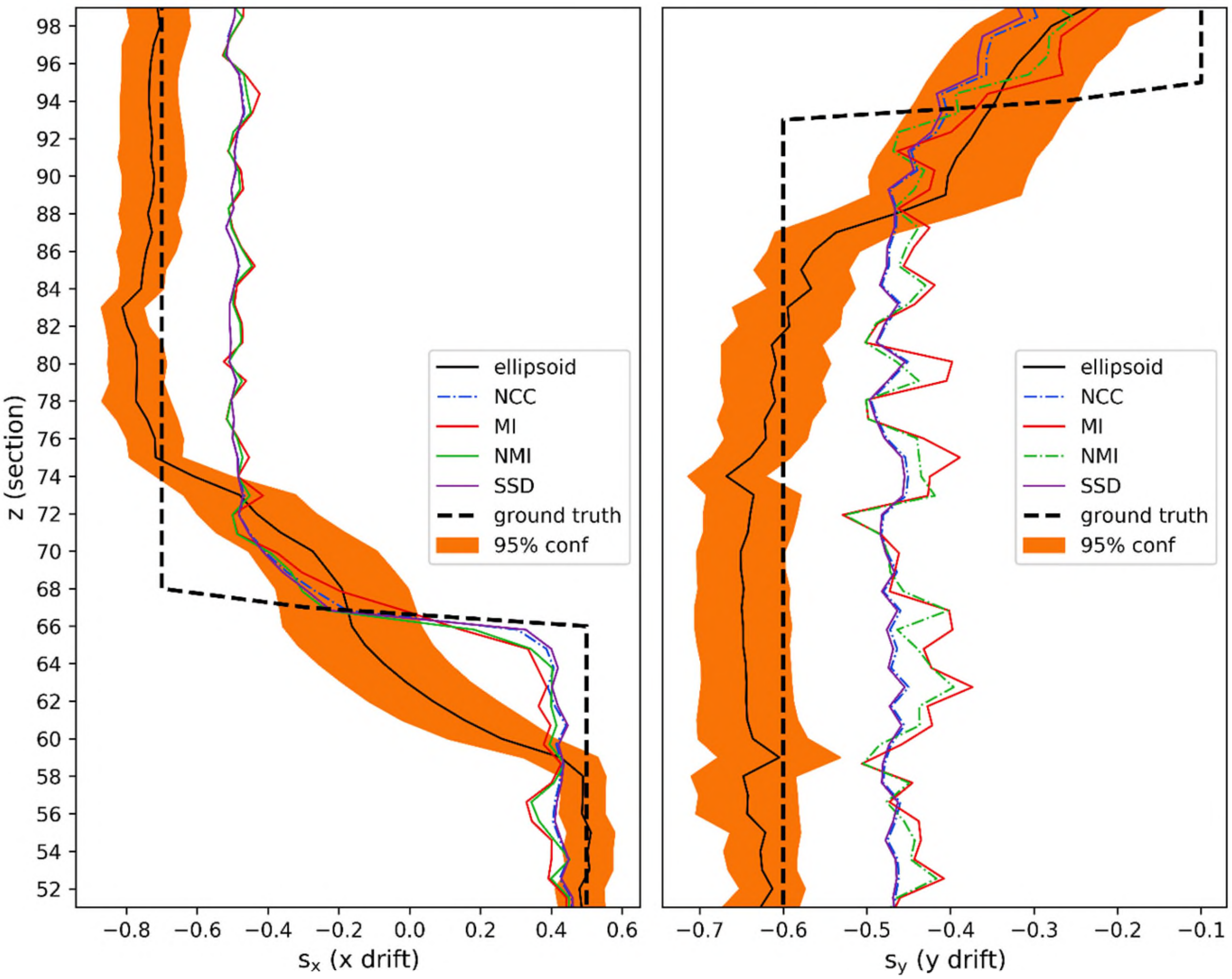

Supplementary Figure 5 | A small subsection of the drift estimate in the synthetic dataset with varying drift. Here the results of the standard approaches are presented without any present smoothing meaning the rapidly varying estimate of the standard registration methods is visible.

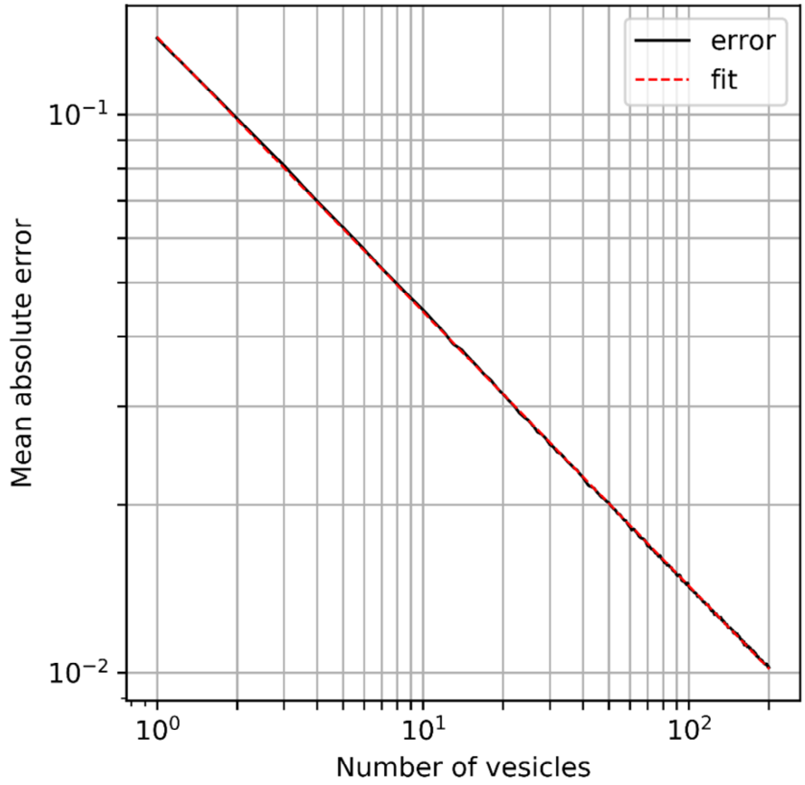

Supplementary Figure 6 | Error plot showing in black how the mean absolute error of the drift estimate is connected to the number of vesicles used in the estimate for a section. This plot was simulated using a bootstrapping approach sampling from the estimated drift components 50000 times for each point on the graph. For each sample, the drift was estimated, and the absolute error measured. This indicates an reciprocal convergence in the error as a function of the number of vesicles. In red is shown the fit ($y = 0.1375x^{-0.4915}$) of the bootstrapping data with.

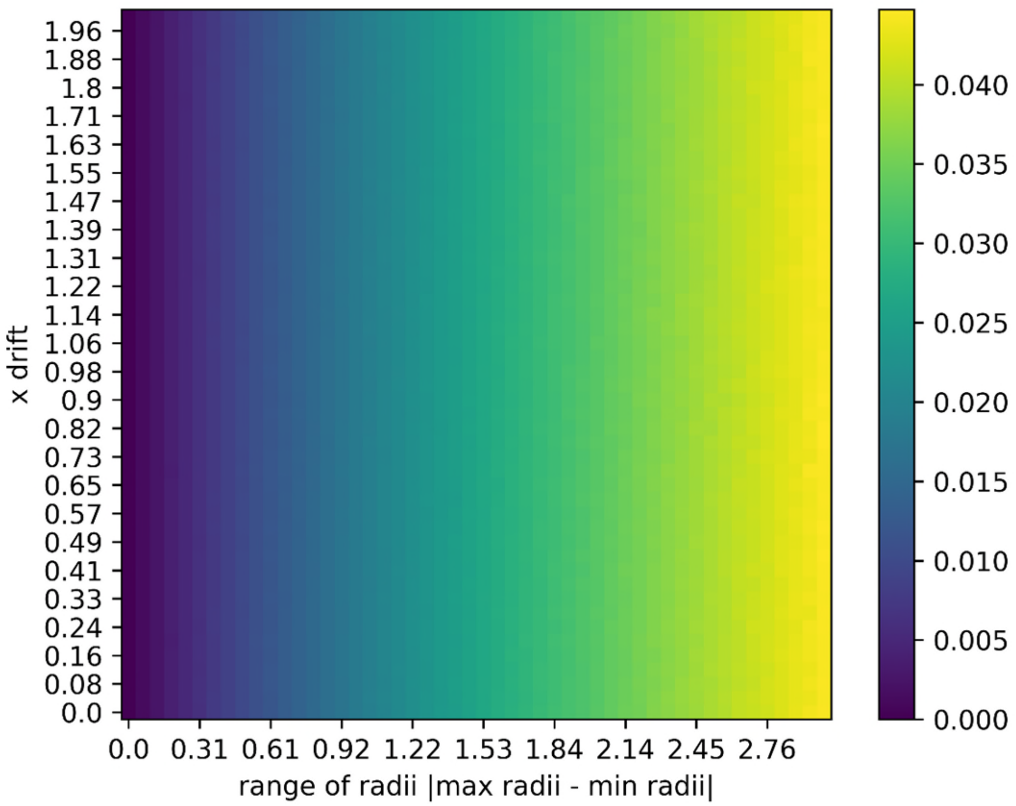

Supplementary Figure 7 | Error plot assessing the interdependence of drift magnitude and radii variation to the mean absolute estimation error. Drift was varied only along the *x*-axis for simplicity since the method is fundamentally independent of the direction of the drift. The radii were varied from 3 to 6 voxels, which is similar to estimates from the FIB-SEM dataset used in this work. The error seems to be independent on the magnitude of the drift.

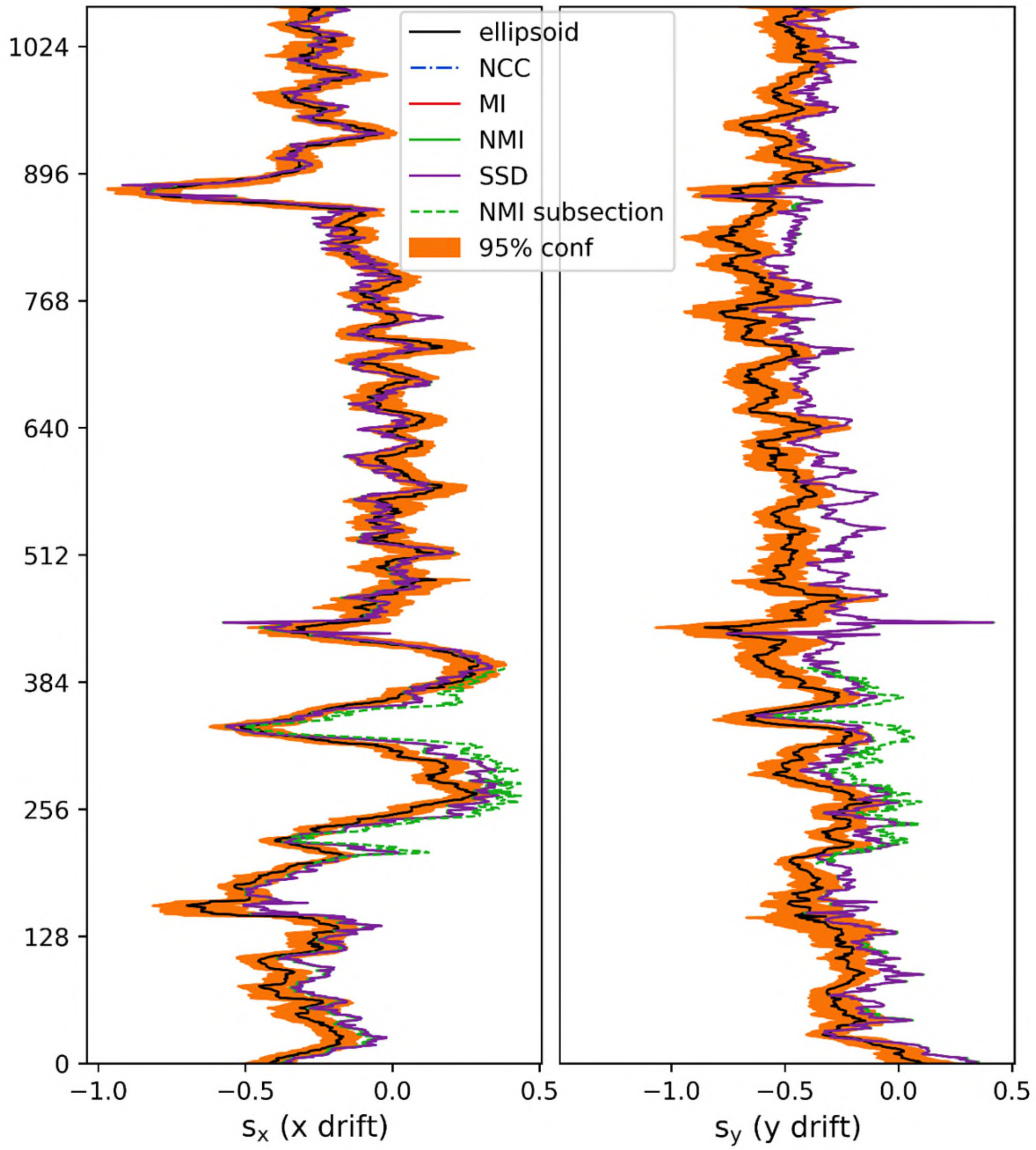

Supplementary Figure 8 | The complete drift estimate comparison in all 1065 sections of the FIB-SEM dataset. All sequences except the one denoted ellipsoid were convolved with a box kernel of size 11 to counter the noise in those approaches to make visual inspection possible. (Left) The drift estimate along the *x*-axis. (right) The drift estimate along the *y*-axis. For both graphs, NCC, MI and SSD coincide. Further, the bias for the standard, global measures appear to be most pronounced along the *y*-axis.

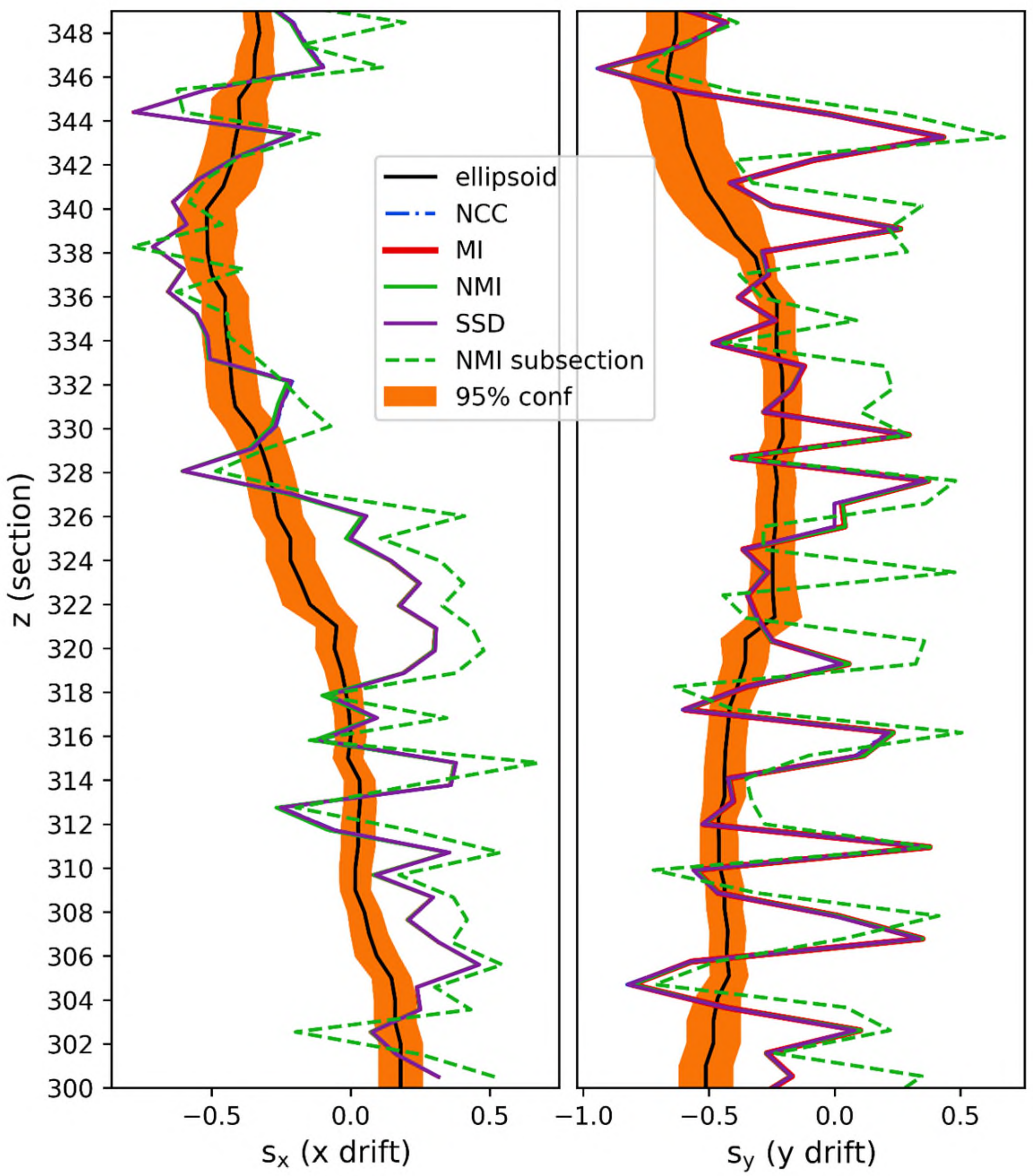

Supplementary Figure 9 | A small subsection of the drift estimate in the real dataset without any smoothing present. Here the rapidly varying drift estimate of the standard registration methods is visible.

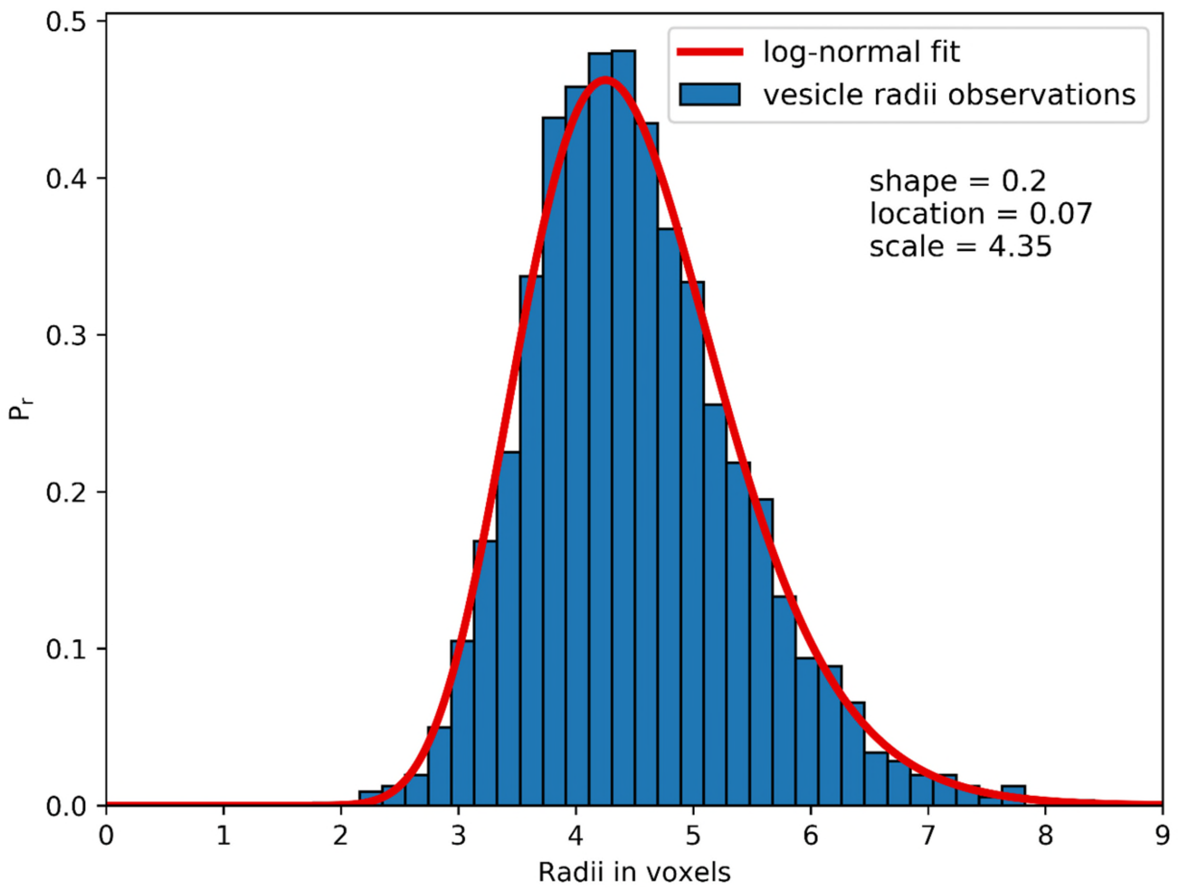

Supplementary Figure 10 | Distribution of ellipsoid radii after drift correction in the FIB-SEM dataset (https://cvlab.epfl.ch/data/data-em/). All radii of all the fitted ellipsoids are used to prevent sorting effects which occurs if largest to smallest radii are assessed separately.

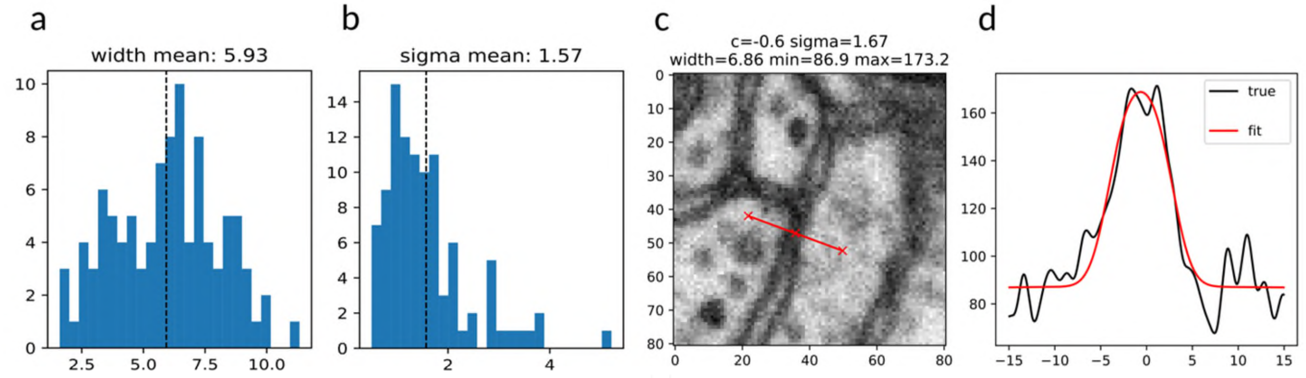

Supplementary Figure 11 | Example of boundary fittings used to estimate the amount of Gaussian blurring in the images. (**a**) Box function width parameters of the membrane fit. (**b**) Gaussian function standard deviation (σ) parameters of the membrane fit. (**c**) A single example of a measurement line for which to fit the blurred box function. (**d**) The (c) corresponding resampling across the line in black along with the fitted Gaussian blurred box function in red.

SUPPLEMENTARY METHODS

To validate our method, we generated multiple synthetic FIB-SEM datasets where the added drift could be systematically controlled. While imitating all aspects of FIB-SEM images is vastly outside the scope of this work, we decided on a subset of requirements the images would have to fulfill. Thus, the dataset was designed with a real FIB-SEM dataset as reference focusing primarily on generating vesicles as they appear there (https://cvlab.epfl.ch/data/data-em/). We modeled vesicles as ellipsoids with varying radii and rotated uniformly at random. Vesicle membrane thickness was chosen to visually match the thickness of the membranes as they appear in the reference dataset. The grayscale values and the contrast were estimated by measuring mean grayscale values in regions in our comparison FIB-SEM image with visually near uniformity for both cytosol and membrane. The radii of the vesicles were generated uniformly at random in the interval from 3 to 6 voxels. Following experiments shows the distribution in real FIB-SEM images are most likely log-normally distributed (**Supplementary Figure 10**). Experiments showed this difference did not have a significant effect on the estimation error.

FIB-SEM images appear blurry because of limitations when imaging at the nano-scale. Modeling this as Gaussian smoothing, we fitted a Gaussian smoothed, box function to membranes of the image. We resampled the image linearly along a line crossing the membrane approximately perpendicular to the membrane. Since the membrane is a 3D structure while we only resampled along a 2D plane, the standard deviation of the Gaussian will be larger when the membrane is not perpendicular to the section direction. Thus, given the resulting distribution (**Supplementary Figure 11**), we conservatively chose to set $\sigma = 1$. FIB-SEM images are also inherently noisy and by our experience appears to be sufficiently well modeled as Gaussian noise. The standard deviation of the noise was estimated by measuring the standard deviation of grayscale values in selected regions in our comparison FIB-SEM image with a near uniform appearance, usually cytosol regions without organelles or visible cytoskeleton.

Synthetic datasets were generated by initializing a volume array of a desired size with the estimated cytosol grayscale value. Vesicle centers were then chosen randomly in the volume. Placement of the vesicle was then attempted several times disregarding those which did overlap with previously placed vesicles. For this purpose, we represent vesicles by the ellipsoid matrix H (**Equation (1)**). Drift was added by multiplying the drift matrix S (**Equation (2))** to each point x. For every point $\mathbf{x}$ in the volume, vesicle membrane grayscale value was set in the volume if $|\mathbf{x}^T S^T H S \mathbf{x} - 1| < 0.25$. After placing a desired number of vesicles, the volume was convolved with a Gaussian kernel to apply the smoothing, whereafter Gaussian noise was added to the images. Values outside the intensity range were clipped.
